# Non-Invasive Delivery of Nano-Emulsified Sesame Oil-Extract of Turmeric Attenuates Lung Inflammation

**DOI:** 10.3390/pharmaceutics12121206

**Published:** 2020-12-11

**Authors:** Sahibzada Tasleem Rasool, Rajasekhar Reddy Alavala, Umasankar Kulandaivelu, Nagaraja Sreeharsha

**Affiliations:** 1 Department of Biomedical Sciences, College of Clinical Pharmacy, King Faisal University, P.O. Box 400, Al-Ahsa 31982, Saudi Arabia; 2 Medicinal Chemistry Research Division, KL College of Pharmacy, KLEF Deemed to be University, Guntur 522502, India; sekhar7.pharm@kluniversity.in (R.R.A.); umasankar@kluniversity.in (U.K.); 3 Department of Pharmaceutical Sciences, College of Clinical Pharmacy, King Faisal University, Al-Ahsa 31982, Saudi Arabia; 4 Department of Pharmaceutics, Vidya Siri College of Pharmacy, Off Sarjapura Road, Bangalore 560035, India

**Keywords:** lung injury, inflammation, allergy, cytokines, turmeric, curcuminoids, sesame oil, nano-emulsion

## Abstract

Turmeric, the golden Indian spice, and the edible oil of sesame seeds are the essential ingredients of Indian food created by ancestors and established the belief of the curative effect of food for many generations. Considering the anti-inflammatory effects of turmeric, we formulated a nano-emulsion of turmeric infused in edible sesame oil, with a globule size of 200–250 nm using high-energy microfluidization. The product with a zeta potential of −11.5 mV showed spherical globules when imaged for scanning and transmission electron microscopy. We explored the anti-inflammatory potential of this edible nano-emulsion in lung inflammation. The lungs are the internal organ most vulnerable to infection, injury, and rapid inflammation from the external environment because of their constant exposure to pollutants, pathogenic microorganisms, and viruses. We evaluated the nano-emulsion for efficacy in ovalbumin-induced lung injury in mice with an oral treatment for two weeks. The therapeutic effect of nano-emulsion of the sesame oil-extract of turmeric was evident from biochemical analysis of bronchoalveolar lavage fluid, lung histopathology, and flow cytometric analysis. The developed nano-emulsion significantly reduced the inflammation and damage to the alveolar network in ovalbumin-injured mice. Significant reduction in the levels of neutrophils and inflammatory cytokines like IL-4, IL-6, and IL-13 in bronchoalveolar lavage fluid was observed in the nano-emulsion-treated group. Leukotriene B4 and IgE were also significantly altered in the treated group, thus suggesting the suitability of the formulation for the treatment of allergy and other inflammatory conditions. The nano-emulsification process potentiated the immunoregulatory effect of turmeric, as observed from the elevated levels of the natural anti-inflammatory cytokine, IL-10. The dietary constituents-based nano-emulsion of spice turmeric helped in scavenging the free radicals in the injured lungs, thus modulating the inflammation pathway. This easily scalable formulation technology approach can therefore serve as a potential noninvasive and safe treatment modality for reducing lung inflammation in lung injury cases.

## 1. Introduction

Lung inflammation, a complex biological process, however, occurs frequently in response to external airborne toxic stimuli and results in substantial cell injury [1]. Aerosolized environmental pollutants, bacteria, viruses, nanoengineered non-biodegradable particles like carbon nanotubes, and ultrafine particles like titanium dioxide and quantum dots cause inflammation of the respiratory airways, as well as deep alveoli, pulmonary edema, and alveolar epithelial cell apoptosis [2]. Inflammation can be acute, as seen in acute lung injury or acute respiratory distress syndrome, causing the rapid migration of neutrophils to the lung production of reactive oxygen species and secreting several enzymes like matrix metalloproteinases, and proteases for the degradation of invading toxins that also cause damage to alveoli [3]. The unusual and out-of-control response by the immune system results in a cytokine storm, as observed in the case of severe acute respiratory syndrome coronavirus (SARS-CoV), which is an unfortunate therapeutic intervention [4]. Exposure to environmental pollutants like cigarette smoke, mycotoxins from fungal spores, and infection by respiratory viruses also cause chronic inflammatory diseases like asthma and chronic obstructive pulmonary diseases [5,6,7]. Steroids are potent anti-inflammatory medicines commonly prescribed for the treatment of lung inflammation and are administered systemically for maximizing the therapeutic effect. Although highly potent, steroidal anti-inflammatory drugs exert high mineralocorticoid effects, necessitating precautions during systemic therapy. Oral delivery, being the most acceptable noninvasive route of administration, is preferred by the patients for the treatment of chronic diseases and will have fewer side effects too. Since time immemorial, the therapeutic effects of plants have been well recognized and are thus a part of therapy, as well as nutrition. From the knowledge of the impact of several dietary nutrients acting on multiple molecular targets, it is possible to design an anti-inflammatory formulation based on common dietary constituents that will offer a unique and traditional pharmacological approach in treating the complex chronic inflammatory lung degenerative diseases. We have designed a nano-emulsion of sesame oil infused with curcuminoids extracted from the pristine and raw form of turmeric, the Golden spice, because nano-emulsion is the safe and effective way of delivery of a poorly soluble and poorly permeable drug to the specific site. Sesame oil is the common dietary constituent used in the Indian food system and has therapeutic potential as an antioxidant and neuroprotective. It is the most common vehicle used for oil-based medications in the Ayurvedic system of Medicine [8]. Turmeric is a rich source of curcuminoids with proven safety and efficacy profiles, especially in the treatment of inflammatory conditions [9]. The novel nano-emulsion of sesame oil extract of turmeric constituents, made by high shear technology, is a safe and effective, bed-to-bench side approach for easy clinical translation and industrial scalability.

## 2. Methods and Methods

### 2.1. Experimental Materials

Turmeric powder, sesame oil, coconut oil, palm oil, olive oil, and sunflower oil were purchased from local vendors. Acetonitrile, aluminum hydroxide, bisdemethoxycurcumin, curcumin, demethoxycurcumin, and ovalbumin (OVA) were obtained from Sigma-Aldrich, Mumbai, India. Absolute ethanol, dexamethasone sodium phosphate (DEX), EDTA solution, Eosin, Formalin, hematoxylin, liquid paraffin, *o*-phosphoric acid, phosphate buffer saline, sodium chloride, and Tween-20 were procured from HiMedia Laboratories, Mumbai, India. ELISA immunoassay kits and Hema 3™ Fixative solutions were purchased from Thermo Fisher Scientific, Waltham, MA, USA. Dulbecco’s modified Eagle’s medium (DMEM), 1X phosphate buffered saline, Trypsin-EDTA, Hanks’ Balanced Salt Solution (HBSS), dimethyl sulfoxide (DMSO), and MTT reagent were purchased from Himedia India. All solvents/chemicals used were of analytical grade.

### 2.2. Extraction of Turmeric in Edible Oils

The mixtures of turmeric powder (10 gm) and five different commonly used edible oils, namely sesame oil, coconut oil, sunflower oil, olive oil, and palm oil (50 mL), were homogenized using a high-speed homogenizer at 10,000 rpm at 70 °C for 15 min (T25, Ultra-Turrax, Ika^®^, Mumbai, India). After cooling to room temperature, the oil portion was separated by filtration under vacuum and stored at room temperature protected from light.

### 2.3. Determination of Curcuminoids in Oils

Stock solution preparation: 0.5 mg/mL standard stock solutions of curcumin, demethoxycurcumin, and bisdemethoxycurcumin were prepared in methanol.

Sample Preparation: Curcuminoids in various edible oils were extracted with methanol (50 mL) and analyzed using Reverse Phase-HPLC after suitable dilutions.

Chromatographic conditions: The chromatogram was developed using an isocratic elution solvent technique with a solvent flow rate of 1 mL/min at room temperature. The HPLC system consisted of a Shimadzu Class LC-10AT VP and LC-20AD pumps connected with an SPD-10A VP UV-Visible detector and on a Cyber Lab C-18 column (250 × 4.0 mm, 5 μ). The components were resolved using a mobile phase of 0.1% *o*-phosphoric acid-acetonitrile (50:50 *v*/*v*) with a flow rate of 1 mL/min at room temperature. Curcumin and its conjugates were detected at 420 nm. The compounds were quantified using HP ChemStation software.

Determination of curcuminoids in samples: 20 µL of various oil samples were used for analysis after suitable extraction and dilution. The amount of curcuminoid present in the samples was determined based on the linear calibration curves and by considering the dilution factor. The amount of the determined curcuminoids was expressed individually in grams per 100 g of crude turmeric powder.

### 2.4. Preparation of Nano-Emulsion and Characterization

The sesame oil extract of turmeric was pre-homogenized with water containing 1% Tween 20 at 12,000 rpm, for 10 min using a high-speed homogenizer (T25, Ultra-Turrax, Ika^®^, Mumbai, India). It was further homogenized in a microfluidizer equipped with a chiller, at 15,000 psi for five cycles (LM 20, Microfluidizer, Microfluidics).

For the determination of globule size, the nano-emulsion sample was placed in a transparent polystyrene cuvette (path length = 1 cm) and placed in a thermostatic sample chamber. The mean globule size and the polydispersity index of the resulting emulsion were determined by photon cross-correlation spectroscopy (Nanophox, Sympatec, Clausthal-Zellerfeld, Germany) with detection at a scattering angle of 90°. Three runs of 60 s were performed at 25 °C and the mean globule size was calculated. The zeta potential was measured on a Zetasizer (ZS 90, Malvern Zeta-sizer, Malvern, UK).

For scanning electron microscopy (SEM), the nano-emulsion (10 μL) was placed on carbon conductive adhesive tape mounted on the specimen stub. The mounted sample was frozen at −190 °C in liquid nitrogen and transferred to the preparation chamber, maintained at −130 °C, and sublimed at −90 °C for 10 min, followed by coating with platinum. It was then transferred to the SEM chamber for viewing at −150 °C with an accelerating voltage of 5.0 kV (JSM-7600F Field Emission Gun (FEG) SEM equipped with Cryo unit (PP3000T) by Quorum).

For transmission electron microscopy (TEM), the nano-emulsion was mounted on a carbon-coated formvar grid, stained with neutralized 2% phosphotungstic acid and dried before imaging.

### 2.5. In Vitro Biocompatibility Testing

The nano-emulsion was evaluated by cell viability in the mouse fibroblast cell line (L929). The cell-line was procured from National Centre for Cell Science (NCCS), Pune, India. The cells were maintained in DMEM (Dulbecco’s modified Eagle medium) and distributed in 96-well plates (1 × 10^4^ cells/well) for 24 h in a 5% CO_2_ atmosphere at 37 °C. After this period, the medium was removed and the adhered cells were treated with formulations (4 dilutions taking 0.2 g/mL of formulation in DMEM as 100%, and further dilution with DMEM to obtain 50%, 25%, and 12.5%, *n* = 6) under the same incubation conditions. Untreated cells were used as controls and considered with 100% cell viability. The viability was determined by MTT assay as described in ISO 10993-5. MTT (1 mg/mL in HBSS) was added to each well of the plate, which was again incubated for 3 h in 5% CO_2_ atmosphere at 37 °C. After this, the medium was aspirated, and the formazan crystals formed were dissolved in DMSO. After 30 min, the optical density was measured on a microplate reader at the wavelength of 570 nm, and the percentage of viability was expressed as the growth rate (%) in comparison to control.

### 2.6. Experimental Animals

Healthy male Swiss-Webster mice, with weights ranging from 20 to 30 g, were selected and divided into six groups, with each group having *n* = 6. They were kept in polypropylene cages at 22 ± 2 °C, maintaining 12 h light and dark cycles. Mice were provided unrestricted water and food access. The authorized diet regime was ad libitum corresponding to the treatment scheme, with morning restrictions of 09–10 AM and evening restrictions of 04–05 PM, periods kept for food supply. The Institutional Animal Ethical Committee approved the protocol for the experimentation under procedure number KLCP/2020/18/005, approved on 20 July 2020.

Out of the six groups, one group was kept as a normal control; Group I—Normal Control (NC) was administered normal saline. The remaining five groups were disease-induced and were treated with different medications as follows. Group II was diseased control and not treated (Ovalbumin-sensitized, OVA), whereas Group III, IV, V, and VI were diseased and treated with dexamethasone (DEX, 0.5 mg/kg), sesame oil in 1% Tween 20 (SO, vehicle control, 1 mL/kg), turmeric powder in 1% Tween 20 (TUR, Formulation control, 20 mg/kg), and nano-emulsion (1 mL/kg), respectively [10,11].

### 2.7. Stimulation of Allergic Symptoms and Treatment

**Figure 1:**
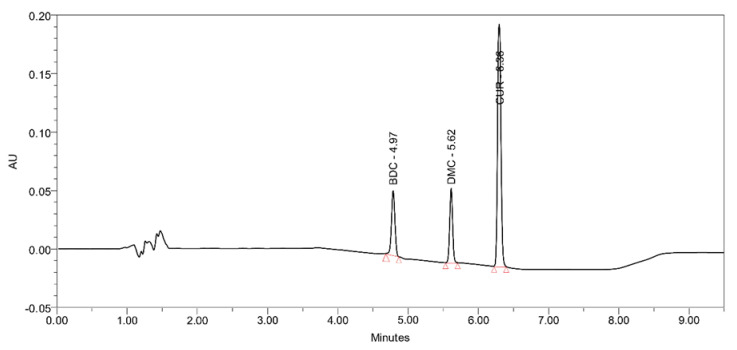
Representative Reverse Phase-HPLC chromatogram of a sesame-oil extract of turmeric showing 3 extracted components curcumin (CUR), demethoxycurcumin (DMC), and bisdemethoxycurcumin (BDC) eluted at 6.36, 5.62, and 4.97 min, respectively.

Ovalbumin (OVA) is capable of triggering allergic asthma according to Nader et al., (2012) [12], indicating an immediate model of hypersensitivity with minimal alterations. The mice in group II to VI were sensitized with an intraperitoneal administration of 0.2 mL OVA suspension (0.01 mg/mL) and aluminum hydroxide (10 mg/mL) in normal saline on day 1 and 10. From day 19 to 24, twice daily for 20 min, the animals were subjected to exposure to aerosolized OVA (3% in normal saline) in a closed chamber connected to a nebulizer (Aeroneb^®^ Lab Nebulizer, Kent Scientific Corporation, Torrington, Connecticut, USA) (Figure 1). The diseased animals in group II to VI were given dexamethasone sodium phosphate, sesame oil in an aqueous solution of Tween-20 1% *v*/*v* solution, turmeric powder in an aqueous solution of Tween-20 1% *v*/*v* solution, and nano-emulsion, respectively. The medications were given orally, 1 h before the challenge from day 12 to 24. Group I was given normal saline. The treatment formulations were administered 1 h before OVA exposure. At the end of the study, the bronchoalveolar lavage fluid (BALF) and blood were collected. Lungs were collected and processed for histopathological evaluation.

### 2.8. BALF Collection and Inflammatory Cell Count

On day 25 of the study, blood samples were collected for IgE and Leukotriene B4 estimation. The animals were tracheostomized for the collection of bronchoalveolar lavage fluid (BAL). Briefly, the lungs were rinsed with 2 × 1 mL washes with ice-cold phosphate buffer solution (pH 7.4) with 1 M EDTA. The obtained BALF was mixed into a single sample. A portion of the resulting solution (0.01 mL) was mixed in a Neubauer chamber with Turk solution (0.39 mL) for complete leukocyte count. The cell suspension (0.02 mL) was applied on slides and coated with Hema 3™ Fixative solutions for differential counts. The total count of each cell type (basophils, acidophil, lymphocytes, granulocyte, and neutrophils) was calculated by obtaining a minimum of 100 cell counts from the slide using an optical microscope with a magnification of 100X to deduce the percentage count of different cell types. The remaining BALF was centrifuged to obtain the clear supernatant and was preserved at −20 °C until further determinations.

### 2.9. Determination of Total Levels of Ovalbumin—Specific IgE and Leukotriene LTB

The blood samples were centrifuged at 5 °C and the plasma separated was used to measure the total IgE, ova-specific IgE, and LTB4 levels using ELISA immunoassay kits (Thermo Fisher Scientific, Pune, Maharashtra, India) as per the instructions of the manufacturer.

### 2.10. Measurement of Inflammatory Cytokines in BALF

The presence and content of various inflammatory mediators including IL-4, IL-6, IL-13, and IL-10 were measured in BALF using ELISA kits (Thermo Fisher Scientific, Pune, Maharashtra, India), as per the instructions of the manufacturer.

### 2.11. Histological Analysis

Animal lungs were washed with a mixture of PBS (pH 7.4) containing EDTA (1 M), harvested, and fixed in 4% formalin in normal saline, approximately 10 h at 4 °C. Later, dehydration was performed using absolute ethanol and coated with liquid paraffin. It was also cut into sections (3 μm) in a microtome and stained for leukocyte infiltrate and edema using hematoxylin and eosin. Under a light microscope using OLYMPUS Stream image analysis, inflammatory cell infiltration, mucus production, and hemorrhagic damage were observed.

### 2.12. Flow Cytometry

Lung mononuclear cells (MNCs) were re-suspended in FACS buffer (Sigma-Aldrich Chemicals, Bengaluru, India) and the cell surface was stained with the antibodies: CD4 violetFluor^®^ 450 (Sigma Aldrich, St. Louis, MO, USA), CD45R (B220) (Sigma Aldrich Chemicals, Bengaluru, India), and Anti-Ly-6G (Gr-1) (Sigma Aldrich Chemicals, Bengarulu, Karnataka, India). Among the CD45+B220+ are B cells, CD3+CD4+ are helper T (TH) cells, and neutrophils are CD45+Gr1+. All cells were examined by flow cytometry (Guava^®^ easyCyte, Merck Millipore, Mumbai, Maharashtra, India) and the results were analyzed using FCS Express (version 7 plus, De Novo Software, Pasadena, CA, USA) [13,14].

### 2.13. Statistical Analysis

Statistical significance was calculated by applying one-way ANOVA using the Student–Newman–Keuls test. The data with values *p* < 0.05 were treated as statistically significant. The statistics of the current study were performed using GraphPad Prism (v7).

## 3. Results

### 3.1. Extraction of Curcuminoids and Estimation of Contents

Turmeric powder was homogenized with various edible oils like palm, sunflower, olive, coconut, and sesame to extract the principal constituent, curcuminoids (CUMs). The content of CUMs in each of the various oils was estimated through RP-HPLC as per Jangle et al., 2013 [15]. The separation of compounds was achieved by HPLC, and curcumin (CUR), demethoxycurcumin (DMC), and bisdemethoxycurcumin (BDC) were eluted at 6.36 ± 0.04, 5.62 ± 0.05, and 4.97 ± 0.03 min (Figure 1), respectively. 

**Figure 2:**
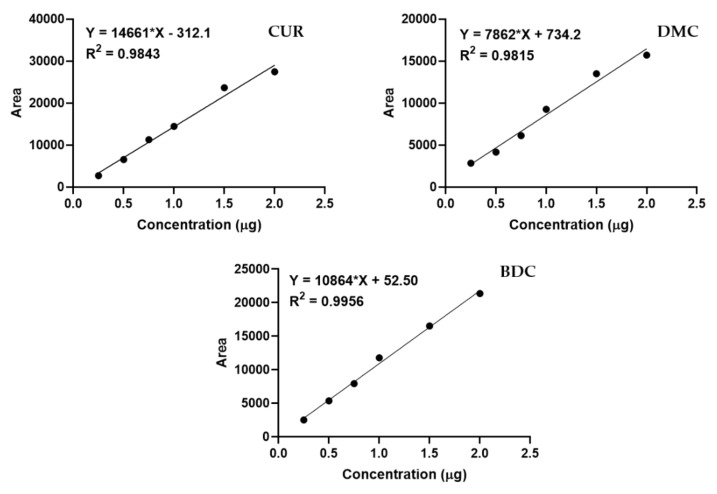
Linearity graphs of individual components of curcuminoids, namely curcumin (CUR), demethoxycurcumin (DMC), and bisdemethoxycurcumin (BDC), used for quantitative analysis of oil extracts of turmeric.

For the quantification of individual components of curcuminoids, individual standard curves were constructed using reference standards, and the linearity graphs are shown in Figure 2.

Curcuminoid content was highest in sesame oil (9.17%), followed by coconut oil (5.22%), olive oil (4.91%), and palm oil (4.78%). However, sunflower oil showed a comparatively poor solubility of curcuminoids (1.98%). The individual percentage compositions of curcuminoids as determined by HPLC based on linearity equations (Figure 2) was found to be 4.07 ± 0.102 (CUR), 3.21 ± 0.033 (DMC), and 1.89 ± 0.058 (BDC).

### 3.2. Characterization of Nano-Emulsion

**Figure 3:**
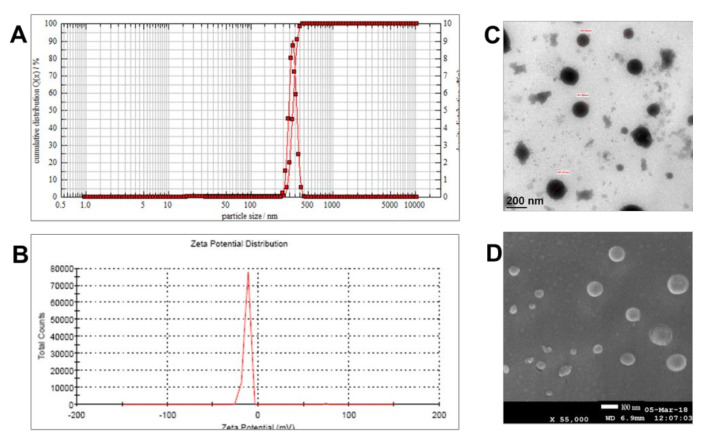
(**A**) Particle size analysis by photon cross-correlation spectroscopy, (**B**) zeta potential, (**C**) transmission electron micrograph (scale bar = 200 nm), and (**D**) scanning electron microscopy (scale bar = 100 nm) image of nano-emulsion of the sesame-oil extract of turmeric.

Photon-cross correlation spectroscopy revealed the nano size of the emulsion globules as 235 ± 7 nm (*n* = 3) with a polydispersity index of 0.21. The single peak in size distribution graph (Figure 3A) shows the monomodal emulsion with a narrow size distribution. The zeta potential of −11.5 ± 2.3 mV (Figure 3B) indicates an anionic charge on the surface of globules, probably due to the ionization of free fatty acids of sesame oil. The spherical nature of the nano-emulsion globule is evident from the transmission electron micrograph (Figure 3C) and Cryo-FEG-scanning electron microscopy image (Figure 3D).

### 3.3. In Vitro Biocompatibility Testing

**Figure 4:**
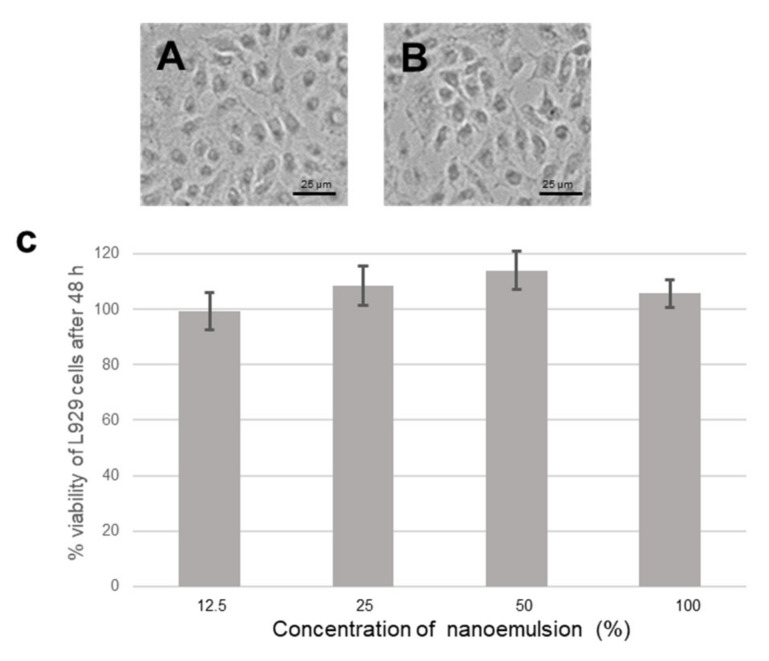
Representative microscopic images of L929 cells treated with nano-emulsion of the sesame-oil extract of turmeric: (**A**) Control and (**B**) treated with formulation 100% (0.2 g/mL). (**C**) Graph showing percentage viability versus concentration of nano-emulsion analyzed using MTT assay.

As observed from microscopic images, the morphology of L929 cells was not altered, because of exposure to the formulation at the highest concentration tested (Figure 4A,B), for 48 h. The L929 cells exhibited more than 98% for all the concentrations tested. Rather, the viability was more than 100%, which is usually observed with lipid-based constituents. This indicates the safety of the product.

### 3.4. Leukocyte Count in BALF

**Figure 5:**
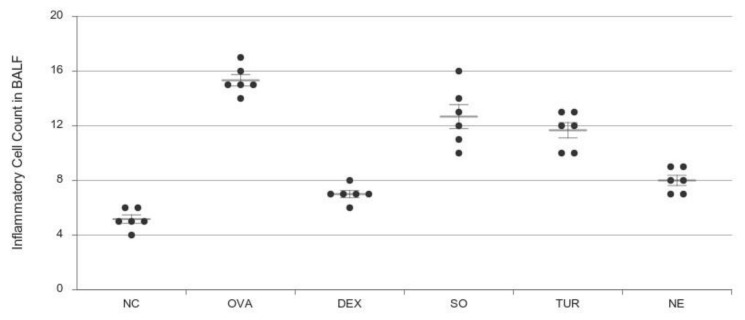
Effect of oral administration of dexamethasone (DEX), vehicle SO, turmeric (TUR), and nano-emulsion on inflammatory cells count in the bronchoalveolar lavage fluid (BALF) of OVA-sensitized mice (i.p. day 1st and 10th) and challenged with OVA (inhalation, from day 19th to 24th). The normal control (NC) received normal saline. The results were expressed as Mean ± SEM (*n* = 6) in cell count × 10^6^ /mL BALF. One-way ANOVA was followed by the Student–Newman–Keuls test. DEX and NE have *p* < 0.001, TER has *p* < 0.01, SO has *p* < 0.05 compared with disease control (OVA), and OVA has *p* < 0.001 compared with NC.

In OVA-induced pulmonary inflammation, significant increases in the total number of neutrophils, eosinophils, and mononuclear cells were observed. The inhibitory effect of sesame oil (SO), TUR, and the developed nano-emulsion were assessed based on the reduction in differential cell count of inflammatory cells. The OVA group showed a significant increase in the number of inflammatory cells observed compared to the normal control group (Figure 5). The total number of leukocytes in each group was expressed as cell count ×10^6^/mL BALF. The inflammation is related to the presence of eosinophils, which is also an indication for asthma. The total count of different cells consisting of eosinophils in BALF was reduced by treatment with SO (13 ± 1.4), TUR (12 ± 1.2), and nano-emulsion (7.8 ± 1.5) × 10^6^/mL BALF. The nano-emulsion group was able to suppress the migration of inflammatory cells to a significant extent (*p* < 0.001). The effect was equivalent to the steroidal drug treatment (DEX). This indicates the potentiation of the anti-inflammatory effect of turmeric by nano-emulsification.

### 3.5. Measurement of Leukotriene B4 (LTB4) and IgE

**Figure 6:**
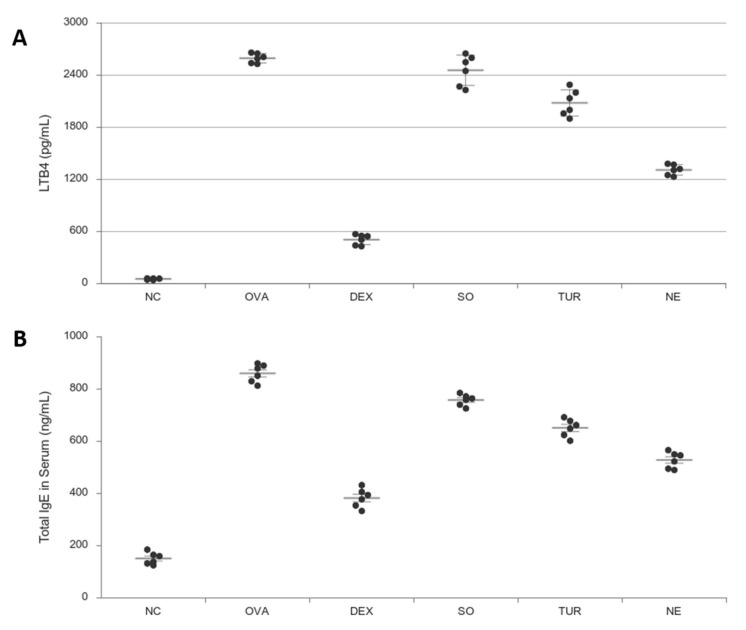
Effect of oral administration of dexamethasone (DEX), vehicle (SO), turmeric (TUR), and nano-emulsion on the levels of (**A**) LTB4 (pg/mL) and (**B**) IgE (ng/mL) in the plasma of OVA-sensitized mice (i.p. 1st and 10th day) and challenged (inhalation, from day 19th to 24th). The results were expressed as Mean ± SEM (*n* = 6). One-way ANOVA was followed by the Student–Newman–Keuls test. DEX and NE has *p* < 0.001, TER has *p* < 0.01, SO has *p* < 0.05 compared with disease control (OVA), and OVA has *p* < 0.001 compared with NC.

LTB4, leukotriene, plays an essential role in the pathogenesis of asthma. LTB4 is a pro-adhesive chemo-attractor for lymphocytes present in the lungs via mastocytes, eosinophils, and alveolar macrophages. The levels of LTB4 (Figure 6A) in the plasma of asthma-induced mice increased (OVA, *p* < 0.001, 2591.34 ± 61.01 pg/mL) when compared to the normal control group (NC 55.25 ± 9.80 pg/mL). In the case of IgE (Figure 6B), in the OVA inflammation-induced lung allergy group, the total IgE levels were significantly higher (850.0 ± 48.3), whereas treatment with DEX, SO, TUR, and nano-emulsion showed a significant lowering of IgE. From both graphs, it is revealed that the dietary constituents’ sesame oil and the spice turmeric have an anti-inflammatory effect; however, the efficacy is enhanced after nano-emulsification.

### 3.6. Determination of Pro-Inflammatory and Anti-Inflammatory Cytokines

**Figure 7:**
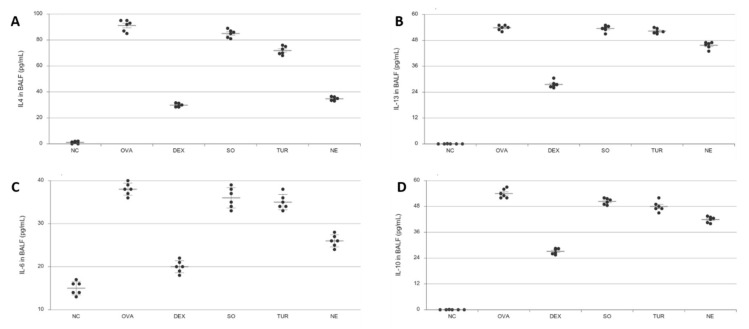
Effect of oral administration of dexamethasone (DEX), vehicle (SO), turmeric (TUR), and nano-emulsion on the levels of (**A**) IL-4 (pg/mL), (**B**) IL-6 (ng/mL), (**C**) IL-13 (pg/mL), and (**D**) IL-10 in the BALF of OVA-sensitized mice (i.p. 1st and 10th day) and challenged (inhalation, from day 19th to 24th). The results were expressed as Mean ± SEM (*n* = 6). One-way ANOVA was followed by Student–Newman–Keuls test. DEX and NE have *p* < 0.001, TER has *p* < 0.01, SO has *p* < 0.05 compared with disease control (OVA), and OVA has *p* < 0.001 compared with NC.

Several cytokines are associated with lung inflammation that are essentially derived from Th2 cells, viz. IL-4, IL-6, and IL-13 were analyzed. IL-10, a pleiotropic and potent anti-inflammatory, immunosuppressive cytokine, was also analyzed to study the immunomodulatory potential of the product. IL-10 is produced by cells of both innate and adaptive immunity including dendritic cells, macrophages, mast cells, natural killer cells, eosinophils, neutrophils, as well as Th2 and Treg cells. The high levels of Th2 pro-inflammatory cytokines are strongly associated with increased airway inflammation in asthma. The nano-emulsion treatment showed a more potentiated anti-inflammatory effect than the corresponding vehicle (SO) and pristine TUR powder (Figure 7). The OVA treatment stimulated the levels of IL-4, IL-6, and IL-13 concentrations in the BALF of the disease control group (OVA). Treatment with nano-emulsion suppressed the inflammation-activated immune system and helped to elevate the levels of the natural anti-inflammatory cytokine, IL-10.

### 3.7. Histopathology of Lungs

**Figure 8:**
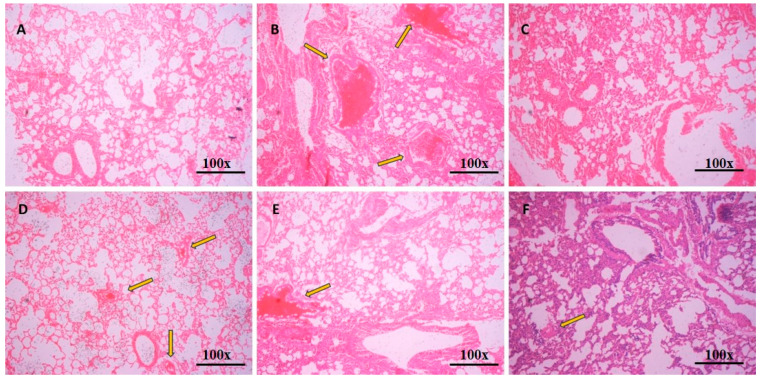
Hematoxylin and eosin-stained lung tissue histopathology images: (**A**) Normal control, (**B**) OVA, disease control, (**C**) DEX, dexamethasone-treated, (**D**) SO, vehicle control sesame oil-treated, (**E**) TUR, turmeric-treated, and (**F**) nano-emulsion-treated group at magnification 100×; scale bar = 100 µm.

Histopathological analysis of lungs showed hemorrhagic injury, and the infiltration of inflammatory cells through the airways. Treatment with sesame oil (SO), pristine turmeric powder (TUR), and the nano-emulsion significantly arrested the tissue damage induced by ovalbumin (OVA) (Figure 8). OVA is frequently used to induce allergic reactions in the lung tissue of mice. Normal control animals (Figure 8A) display a lack of cellular penetration in the peribronchial region and perivascular spaces. Ovalbumin-sensitized and challenged mice showed inflammatory cells infiltrating the peribronchial area and perivascular spaces in addition to the growth of mucus in the bronchial lumen (Figure 8B). Animals treated with dexamethasone (DEX), formulation vehicle sesame oil (SO), formulation control, pristine turmeric powder (TUR), and nano-emulsion exhibited reduced cellular infiltrate in the peribronchial region and perivascular spaces.

## 4. Discussion

Oxidative stress and associated inflammation in the lung and the circulation in response to exposure to air pollution, tobacco smoke, infection, or potentially toxic non-biodegradable nanoparticles are the leading pathogenic processes responsible for either acute or chronic lung injury [16]. Several dietary constituents contribute to antioxidant/oxidant and inflammatory status in lung injury. In general, the population has diets with lower fruit and vegetable intake; thus, the consumption of fewer antioxidants leads to more vulnerability to impaired lung function and the risk of having lung injuries [17]. There is not a single healthy diet that can act as a magic bullet for pulmonary functioning. Indian, Chinese, Mediterranean, or Nepalese foods may have therapeutic values against certain diseases due to their abundance of antioxidants. Most of the activities associated with foods were related to the compounds present in the raw materials used for their preparation, such as spices like turmeric and other natural products [18]. Turmeric is a spice and is known as the main ingredient in most of the spicy curries in India and other parts of Asia, and has been extensively utilized for a long time in various diseases. Curcumin is the main bioactive component in turmeric that has powerful anti-inflammatory and antioxidant effects [19,20,21,22]. The acute brain inflammation and its damage, leading to long-term memory loss caused by lipopolysaccharides, can be significantly reduced by treatment with turmeric in mice [19]. It has also been demonstrated to alleviate ventilator-stimulated injury and inflammation in the lungs via inhibition of NF-κB in rats [20]. Curcumin nano-emulsion has been explored as a safe and effective formulation to prevent tumor reincidence and metastasis [23]. Curcuminoid extracts and nano-emulsion prepared from *Curcuma longa* inhibited the growth of lung cancer cells A549 and H460, thus proving the potential of curcumin nano-emulsion for lung delivery [24].

In contrast to isolated phytoconstituents, the medicinal extract components often work synergistically to produce their therapeutic effects. However, innovative strategies are required to present these extracts in an acceptable and easily scalable approach without altering the functionality. Turmeric has poor bioavailability (around 1%), so it requires extremely high doses for it to have any appreciable effects [18]. The principal constituent of turmeric is curcuminoids, which exhibit antioxidant properties due to the inhibition of reactive oxygen species like hydrogen peroxide superoxide and nitric oxide radicals [25,26].

Sesame oil is an edible oil that is frequently used as an adjuvant during food preparation in the Mediterranean, Indian, or Nepalese areas along with some parts of Asia. The oil contains mainly fatty acids (eicosanoic, linoleic, linolenic, oleic, palmitic, palmitoleic, and stearic acids), lignans, and antioxidants (γ-tocopherol) [27,28]. Sesame oil has already been proven to possess potent anti-inflammatory activities [29], capable of treating heavy metal poisoning [30] along with being able to reduce pulmonary inflammation by inhibiting IgE levels [10].

The effect of nanoemulsifying the sesame oil-extract of turmeric, on influencing positive outcomes in allergic asthma has not been previously examined. In this study, the potential protective activity of the nano-emulsion of dietary constituents on lung inflammation and edema was investigated by employing an experimental allergic asthma mouse model. Nano-emulsion was found to reduce pulmonary edema by inhibiting pulmonary inflammation in our animal model. Pulmonary edema may not be the primary characteristic of asthma; it may occur following acute aggravation of an asthmatic condition [31]. Pro-inflammatory cytokines (IL-1 and IL-6) are known to intensify the inflammatory cascade and to improve vascular penetrability, which can lead to the formation of pulmonary edema in allergic asthma [32,33,34].

It is known that the inflammatory cascade plays a significant role in the development of chronic diseases. In this study, pro-inflammatory cytokine production was found to be substantially decreased by the nano-emulsion of turmeric extract. It reduced inflammation and pulmonary edema by inhibiting the infiltration of polymorphonuclear leukocytes. By inhibiting inflammatory cytokines, chemokines, and enzymes, nano-emulsion decreased neutrophil invasion, with decreases in associated pulmonary edema, and inflammation [35]. A protective effect from pulmonary edema in different models is indicative of Leucocyte infiltration [36,37]. The pathways of MAPK, NF-kB, and IL-1β, IL-6, and TNF-α can also be minimized by treatment with curcumin [38,39]. In BALF and neutrophil infiltration in the lung, treatment with nano-emulsion decreased cell counts. It has been supported with the flow cytometric data, where the nano-emulsion has reduced the elevated number of immune cells infiltrating the lung tissue (Supplement Materials Figures S1 and S2). The effect was observed on the B cells, neutrophils, and T_H_ cells, which are major contributors for the formation of inflammatory mediators. The treatment of TUR and SO alone has not given significant results on the reduction in the percentage of immune cells in lungs. We suggest that sesame oil-extract of turmeric in nanoemulsified form can be used to prevent allergic pulmonary inflammation associated with asthma by inhibiting the infiltration of leukocytes, especially neutrophils.

The anti-inflammatory activities of curcuminoids in several studies have shown that curcumin was able to regulate receptor levels of Notch1/2 and GATA3 in acute allergic asthma caused by inflammation in BALB/c mice [40,41]. Nano-emulsion has been shown to lower IgE levels in mice sensitized with OVA, and thus serves as a possible new treatment for allergic asthma. In allergic asthma pathogenesis, IgE plays a key role with systemic IgE levels directly proportional to chronic asthma, even though IgE had not been previously considered as a clinical biomarker [42]. Antibodies may control IgE, which displays a strong beneficial influence in allergic asthma in both animals and humans [24]. In addition, curcuminoids minimize the inflammation due to allergic reactions in the asthma animal model by controlling the Treg to Th17 equilibrium by increasing the Treg cell concentration [43].

The developed nano-emulsion has potential beneficial biological effects in reversing the oxidative lung damage. Although the technology has promising therapeutic effects, quality consistency with respect to natural products is a concern and needs to be optimized for successful clinical translation. Acute toxicity studies as per OECD guidelines and dose optimization studies for effective tailoring of the dose for human application will help to move a step ahead in commercialization.

## 5. Conclusions

All over the world, lung inflammation, either chronic or acute, is the leading cause of morbidity and mortality. Steroidal anti-inflammatory drugs are of choice for the rapid relief of inflammation and for arresting the further cascade of inflammatory reaction leading to tissue damage. However, numerous side effects associated with steroidal therapy necessitates new safe drug development. However, the low success rate of new drug discovery requires a paradigm shift for innovative drug development strategies, and thus getting inspiration from natural products for effective treatment of disease needs to be appreciated. The use of nanotechnology alters the physicochemical and biological properties of natural products, making them potent synthetic drugs. In the present study, the nanoemulsification process using routinely consumed food materials potentiated the immunoregulatory effect of turmeric extracted in sesame oil as observed from elevated levels of the natural anti-inflammatory cytokine, IL-10. Being well-established dietary constituents, the product is digestible, biodegradable, and safe. The dietary constituents-based nano-emulsion of spice turmeric helped in scavenging the free radicals in the injured lungs, thus modulating the inflammation pathway. This easily scalable and food constituents-based formulation technology can therefore serve as a potentially safe and noninvasive treatment modality for reducing lung inflammation in lung injury cases. The multipronged therapeutic approach proved as a platform technology can be easily adapted to other chronic inflammatory diseases too.

## Supplementary Materials

The following are available online at https://www.mdpi.com/1999-4923/12/12/1206/s1. Figure S1: The flowcytometry diagram of immune cells infiltration in lung tissue. (A) B cells (CD45+B220+), (B) neutrophils (CD45+Gr1+), and (C) helper T (TH) cells (D3+CD4+) in different treatment groups namely NC (Normal control), Ovalbumin sensitized (OVA), Dexamethasone (DEX), vehicle SO, TUR, and nano-emulsion (NE)**.** Figure S2: Nano-emulsion reduced the population of various immune cells in the lungs of the OVA sensitized mice. (A) B cells (%); (B) Neutrophils (%) and (C) TH cells (%) in different treatment groups namely, NC (Normal control), Ovalbumin sensitized (OVA), Dexamethasone (DEX), vehicle SO, TUR, and nano-emulsion (NE).
