# Correction: Rasool et al. Non-Invasive Delivery of Nano-Emulsified Sesame Oil-Extract of Turmeric Attenuates Lung Inflammation. *Pharmaceutics* 2020, *12*, 1206

**DOI:** 10.3390/pharmaceutics17050582

**Published:** 2025-04-29

**Authors:** Sahibzada Tasleem Rasool, Rajasekhar Reddy Alavala, Umasankar Kulandaivelu, Nagaraja Sreeharsha

**Affiliations:** 1 Department of Biomedical Sciences, College of Clinical Pharmacy, King Faisal University, P.O. Box 400, Al-Ahsa 31982, Saudi Arabia; 2 Medicinal Chemistry Research Division, KL College of Pharmacy, KLEF Deemed to be University, Guntur 522502, India; sekhar7.pharm@kluniversity.in (R.R.A.); umasankar@kluniversity.in (U.K.); 3 Department of Pharmaceutical Sciences, College of Clinical Pharmacy, King Faisal University, Al-Ahsa 31982, Saudi Arabia; 4 Department of Pharmaceutics, Vidya Siri College of Pharmacy, Off Sarjapura Road, Bangalore 560035, India

## Error in Figure

In the original publication, there was a mistake in Figure 8 as published [1]. It was found that there was a modest similarity in the histopathology images of different models, where some portions of image A, C and E seems to be similar. The corrected Figure 8 appears below. The authors state that the scientific conclusions are unaffected. This correction was approved by the Academic Editor. The original publication has also been updated.

## Figures and Tables

**Figure 8:**
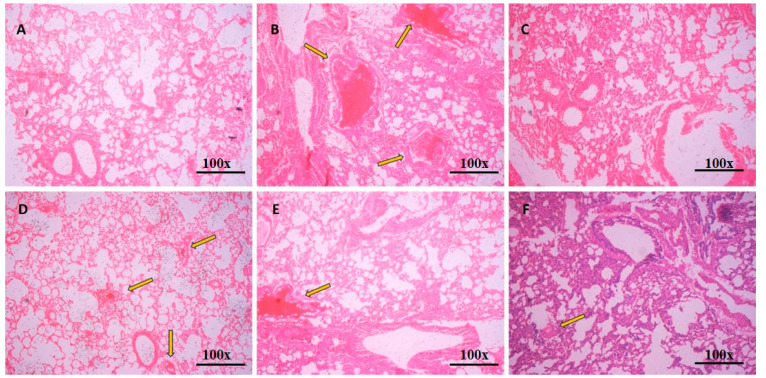
Hematoxylin and eosin-stained lung tissue histopathology images: (**A**) Normal control, (**B**) OVA, disease control, (**C**) DEX, dexamethasone-treated, (**D**) SO, vehicle control sesame oil-treated, (**E**) TUR, turmeric-treated, and (**F**) nano-emulsion-treated group at magnification 100×; scale bar = 100 µm.
