# Supplementary material for: Non-Invasive Delivery of Nano-Emulsified Sesame Oil-Extract of Turmeric Attenuates Lung Inflammation
Source: Pharmaceutics. 2020 Dec 11;12(12):1206. doi: 10.3390/pharmaceutics12121206 (PMC7764660; doi:10.3390/pharmaceutics12121206)
Supplement: Supplementary file 1 [file pharmaceutics-12-01206-s001.pdf]

Supplementary Data

Non-invasive delivery of nano-emulsified sesame oil-extract of turmeric attenuates lung inflammation

Sahibzada Tasleem Rasool [1]*, Rajasekhar Reddy Alavala [2], Umasankar Kulandaivelu [2], Nagaraja Sreeharsha[3, 4]*

[1] Department of Biomedical Sciences, College of Clinical Pharmacy, King Faisal University, P.O. Box 400,0 Al-Ahsa, 31982, Kingdom of Saudi Arabia; srasool@kfu.edu.sa (S.T.R)

[2] Medicinal Chemistry Research Division, K L College of Pharmacy, K L E F Deemed to be University, Guntur, Andhra Pradesh, India-522 502; sekhar7.pharm@kluniversity.in (R.R.A); umasankar@kluniversity.in (U.K)

[3] Department of Pharmaceutical Sciences, College of Clinical Pharmacy, King Faisal University, Al-Ahsa-31982, Saudi Arabia; sharsha@kfu.edu.sa (N.S.)

[4] Department of Pharmaceutics, Vidya Siri College of Pharmacy, Off Sarjapura Road, Bangalore - 560035, Karnataka, India.

* Correspondence: srasool@kfu.edu.sa; shasha@kfu.edu.sa

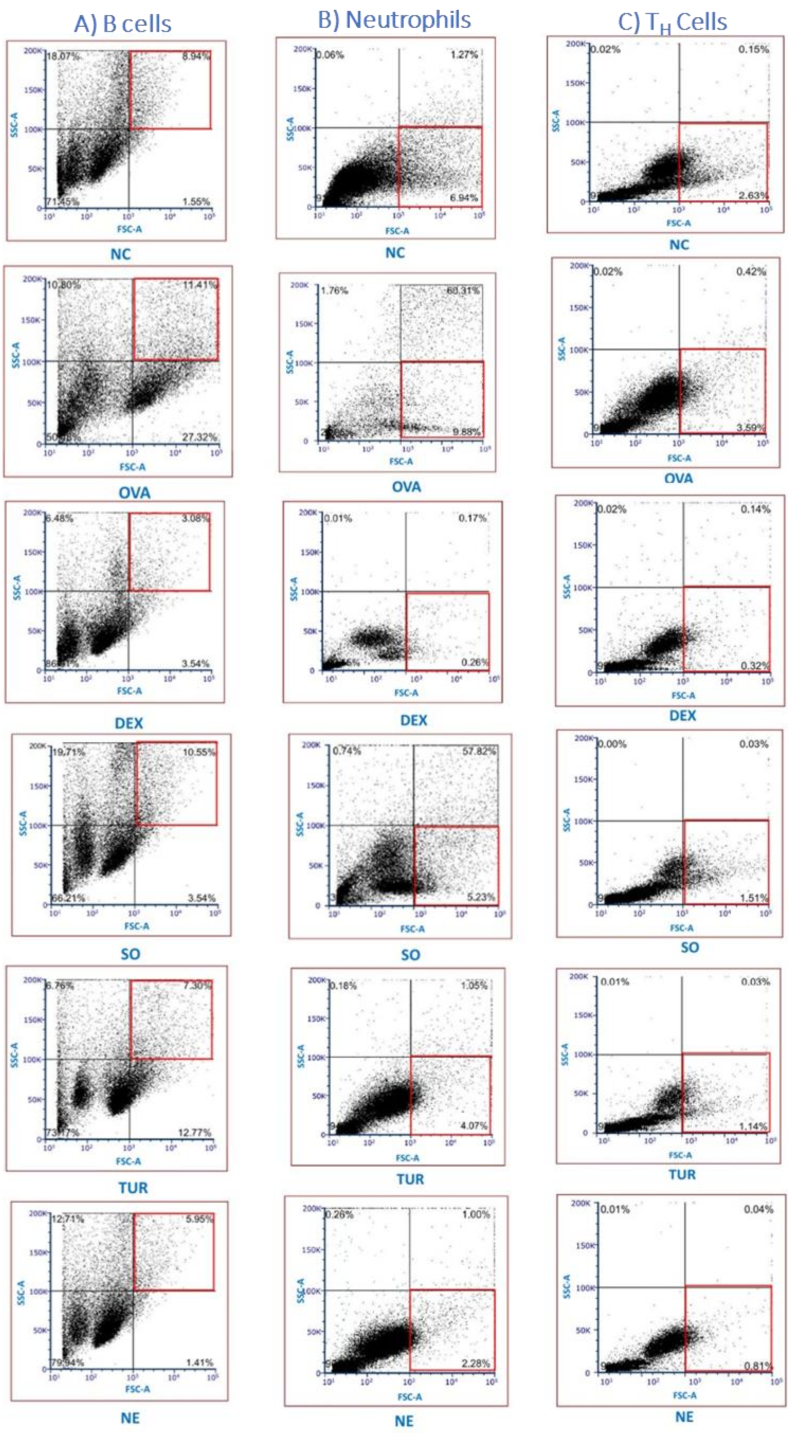

Figure S1: The flowcytometry diagram of immune cells infiltration in lung tissue. A) B cells (CD45+B220+), B) neutrophils (CD45+Gr1+), and C) helper T (T_H) cells (D3+CD4+) in different treatment groups namely NC (Normal control), Ovalbumin sensitized (OVA), Dexamethasone (DEX), vehicle SO, TUR, and nano-emulsion (NE)

Flow cytometric analysis of BALF: Nano emulsion decreased the infiltration of immune cells in the lung:

In the case of B cells, the control group population of cells is 8.94 %, which was increased by the OVA treatment to 11.41 % (Figure S1A, S2A). The treatment with TUR and NE has resulted in significant reduction of cell infiltration to 7.3 and 5.95 % respectively. Whereas the DEX has given 3.08 % of B cell infiltration and SO treatment had no significant effect on reduction in cell count (10.55 %).

The number neutrophils found in the control group was 6.94 %, which was increased to 9.88 % by the treatment of OVA (Figure S1B and S2B). The cell count decreased to 4.07 and 2.28 % with the treatment of TUR and NE, respectively. The standard DEX inhibition was found to be 0.26 % and the SO group resulted 5.23 % neutrophils infiltration in the lungs.

Of the overall number of T_H cells (2.6 %) in the control group, the OVA treatment has risen to 3.59 % and decreased to 1.14 % by TUR (Figure S1C and S2C). Whereas the nano-emulsion has decreased the T_H cells to 0.81 %, which is comparable with standard DEX of 0.32 %. The SO treatment resulted 1.51 % of infiltration of T_H cells in the lung tissue.

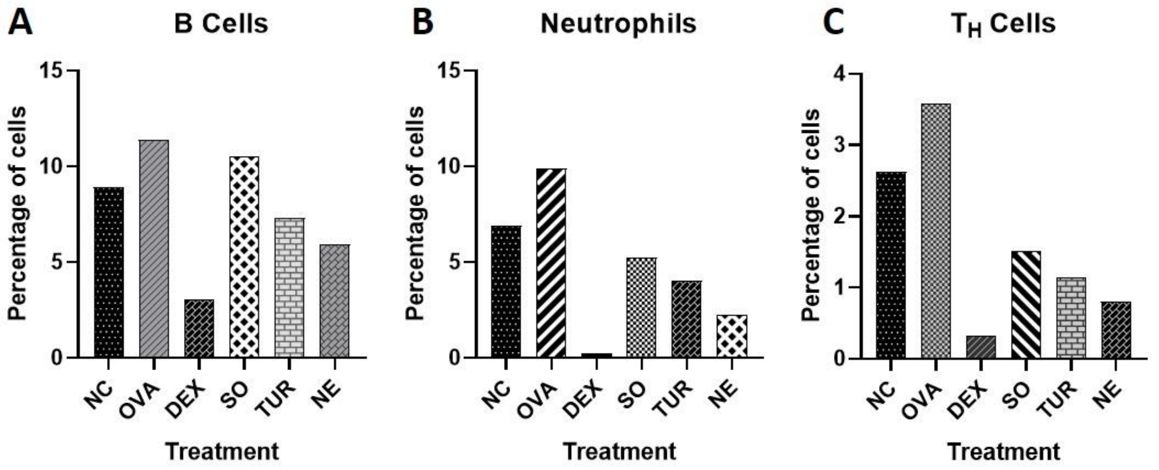

Figure S2: Nano-emulsion reduced the population of various immune cells in the lungs of the OVA sensitized mice. (A) B cells (%); (B) Neutrophils (%) and (C) T_H cells (%) in different treatment groups namely, NC (Normal control), Ovalbumin sensitized (OVA), Dexamethasone (DEX), vehicle SO, TUR, and nano-emulsion (NE).
